# RETRACTION: Effects of Thoracoscopic Lobectomy on Surgical Wound Infection in Patients With Lung Cancer: A Meta‐Analysis

**DOI:** 10.1111/iwj.70441

**Published:** 2025-03-26

## Abstract

**RETRACTION:** YangG. , RenM.‐Z. , XinX.‐B. , NiuY.‐J. , and WangT. , “Effects of Thoracoscopic Lobectomy on Surgical Wound Infection in Patients With Lung Cancer: A Meta‐Analysis,” International Wound Journal 21, no. 2 (2024): e14684, 10.1111/iwj.14684.PMC1085092442052947

The above article, published online on 07 February 2024, in Wiley Online Library (http://onlinelibrary.wiley.com/), has been retracted by agreement between the journal Editor in Chief, Professor Keith Harding; and John Wiley & Sons Ltd. Following an investigation by the publisher, all parties have concluded that this article was accepted solely on the basis of a compromised peer review process. The editors have therefore decided to retract the article. The authors did not respond to our notice regarding the retraction.

**RETRACTION:** G. Yang , M.‐Z. Ren , X.‐B. Xin , Y.‐J. Niu , and T. Wang , “Effects of Thoracoscopic Lobectomy on Surgical Wound Infection in Patients With Lung Cancer: A Meta‐Analysis,” International Wound Journal 21, no. 2 (2024): e14684, 10.1111/iwj.14684.PMC1085092442052947

The above article, published online on 07 February 2024, in Wiley Online Library (http://onlinelibrary.wiley.com/), has been retracted by agreement between the journal Editor in Chief, Professor Keith Harding; and John Wiley & Sons Ltd. Following an investigation by the publisher, all parties have concluded that this article was accepted solely on the basis of a compromised peer review process. The editors have therefore decided to retract the article. The authors did not respond to our notice regarding the retraction.

